# Stable alterations of CD44 isoform expression in prostate cancer cells decrease invasion and growth and alter ligand binding and chemosensitivity

**DOI:** 10.1186/1471-2407-10-16

**Published:** 2010-01-14

**Authors:** Kui Yang, Yaqiong Tang, Gabriel K Habermehl, Kenneth A Iczkowski

**Affiliations:** 1 Department of Pathology, University of Colorado Denver Health Science Center, Aurora, Colorado, USA

## Abstract

**Background:**

Dysregulated CD44 expression characterizes most human cancers, including prostate cancer (PCa). PCa loses expression of CD44 standard (CD44s) that is present in benign epithelium, and overexpresses the novel splice variant isoform, CD44v7-10.

**Methods:**

Using retroviral gene delivery to PC-3M PCa cells, we expressed luciferase-only, enforced CD44s re-expression as a fusion protein with luciferase at its C-terminus or as a protein separate from luciferase, or knocked down CD44v7-10 by RNAi. Invasion, migration, proliferation, soft agar colony formation, adhesion, Docetaxel sensitivity, and xenograft growth assays were carried out. Expression responses of merlin, a CD44 binding partner, and growth-permissive phospho-merlin, were assessed by western blot.

**Results:**

Compared to luciferase-only PC-3M cells, all three treatments reduced invasion and migration. Growth and soft agar colony formation were reduced only by re-expression of CD44s as a separate or fusion protein but not CD44v7-10 RNAi. Hyaluronan and osteopontin binding were greatly strengthened by CD44s expression as a separate protein, but not a fusion protein. CD44v7-10 RNAi in PC-3M cells caused marked sensitization to Docetaxel; the two CD44s re-expression approaches caused minimal sensitization. In limited numbers of mouse subcutaneous xenografts, all three alterations produced only nonsignificant trends toward slower growth compared with luciferase-only controls. The expression of CD44s as a separate protein, but not a fusion protein, caused emergence of a strongly-expressed, hypophosphorylated species of phospho-merlin.

**Conclusion:**

Stable re-expression of CD44s reduces PCa growth and invasion in vitro, and possibly in vivo, suggesting CD44 alterations have potential as gene therapy. When the C-terminus of CD44s is fused to another protein, most phenotypic effects are lessened, particularly hyaluronan adhesion. Finally, CD44v7-10, although it was not functionally significant for growth, may be a target for chemosensitization.

## Background

About 30% of cases of prostate cancer (PCa) undergo transition from quiescent to aggressive. In this transition, altered expression of adhesion glycoproteins such as CD44 occurs allowing tumor cells to detach, interact with proteins that digest stromal matrix, migrate through matrix, and intravasate into lymphovascular channels. CD44 is a transmembrane molecule encoded by an alternately spliced gene. The standard (**CD44s**) isoform is ubiquitous, but inclusion of one or more of 10 variant (**v**) exons lengthens the extracellular stem, producing tissue-specific (**CD44v**) isoforms. CD44 is involved in multiple cellular functions. Its N-terminus enables cell-cell adhesion and binds hyaluronan and other matrix ligands, while the C-terminus links the cell's membrane to actin and ankyrin in the cytoskeleton, modulating shape and motility.

In prior work, we isolated RNA from clinical PCa tissues and discovered that expression of CD44v7-10 variant isoform constitutes a unique PCa signature, consistently expressed in primary and metastatic PCa. Androgen-independent PCa cell lines also strongly expressed it[1-3]. Interference against CD44v7-10 caused a 69% reduction in invasion index compared to untreated control cells[3] and altered ligand-binding affinities[4]. Moreover, PCa loses the splicing ability to produce the standard isoform expressed in benign prostate[3,5,6], and certain variants other than CD44 v7-10[1,7]. Stable, virally altered CD44 expression has not been tested in any system. We enacted transient CD44v7-10 RNAi in PC-3 cell variants using the plasmid U6pBS which lacks a drug-resistance selection gene[3], and later using pTracer with drug resistance and GFP signal genes[4,8], and produced several phenotypic changes. Others have also enacted transient plasmid transfection of CD44s into PC-3 cells, causing reduced growth *in vitro *and tumorigenicity in mouse xenografts[9].

Here, we perform the first comprehensive assessment of two types of stable, retroviral CD44 alterations on PCa cells and their functional effects. First, two CD44s re-expression sequences were created--one translating to a fusion protein comprising CD44s with luciferase at its cytoplasmic, C-terminus, and the other expressing these as two separate proteins. Second, a CD44v7-10 RNAi sequence was expressed. Their potential for human gene therapy was tested by in vitro invasion, attached growth, anchorage-independent growth, and chemosensitization, and by creating subcutaneous xenografts. Dephosphorylated merlin binds CD44 and inhibits growth, whereas merlin is inactivated by phosphorylation to a growth-permissive state. Because one of the two CD44s re-expression transfectants has a free cytoplasmic -COOH tail and the other a bound tail, we assessed whether merlin, a binding partner of CD44's tail, may be sterically hindered from interaction with luciferase-fused CD44s.

## Methods

### Cell lines

Benign BPH-1 and 293T cells were from American Type Culture Collection (Manassas, VA). PC-3M cells, a metastasis-derived variant of PC-3, were from Dr. I. J. Fidler, M.D. Anderson Cancer Center, Houston, TX. The culture medium for PC-3M cells was RPMI 1640 (Invitrogen, Carlsbad, CA) with 10% fetal calf serum (FCS) and antibiotics. 293T cells were in Dulbecco's modified Eagle medium with FCS. Cells were grown in 5% CO_2 _incubation at 37°C. For cell set-up, cells in a flask were trypsinized, medium with serum was added to neutralize trypsin, and cells were stained in Trypan blue and counted by grid method[4].

### Viral (Stable) CD44 Transfectants; Merlin Transfectants

**Figure 1:**
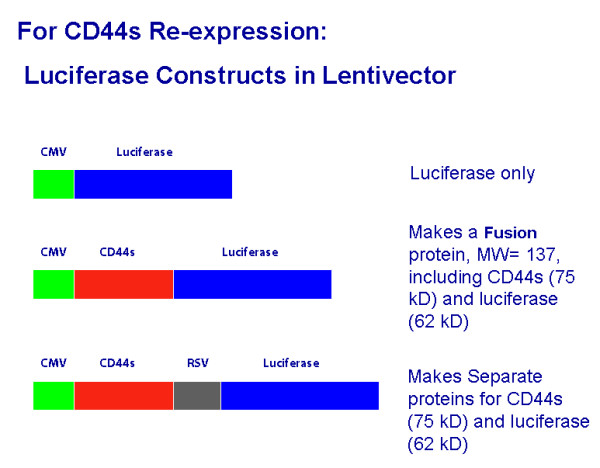
**Constructs used for CD44s re-expression in Lentivector. **CD44s was expressed as a fusion protein with luciferase, or as a separate protein. CD44s in human prostate includes exons 1-5 and 16-18, with a small portion of 20, according to our sequencing[3].

Three constructs were made in Lentivector pLEX-MCS (Open Biosystems, Huntsville, AL), which contains the cytomegalovirus promoter, associated sequences, and puromycin resistance gene. These were Lenti-luciferase, Lenti-CD44s-luciferase, and Lenti-CD44s-RSV-luciferase (Figure 1). Lenti-luciferase was made by inserting the luciferase fragment from a plasmid (gift of Dr. J. Shen, Department of Radiation Oncology, Univ. of Colorado Denver) behind CMV promoter. A "Fusion" construct, Lenti-CD44s-luciferase, was then made by PCR-amplifying the CD44s fragment from our pTracer construct[7] and subcloning it into Lenti-luciferase. The fusion protein expressed comprises CD44s with luciferase at its C-terminal end. The "Separate" construct, Lenti-CD44s-RSV-luciferase, was made by inserting an RSV promoter between CD44s and luciferase sequences to drive luciferase expression. This construct expresses separate CD44s and luciferase proteins. PC-3M cells were infected with the 3 respective constructs to yield PC-3M-luciferase cells, PC-3M-CD44s-Fusion cells, or PC-3M-CD44s-Separate cells. Cells were grown in 1 μg/mL puromycin medium. Persistent expression was confirmed by adding luciferin substrate and imaging the flask on an IVIS 200 system (Xenogen, Alameda, CA), and by western blot analysis prior to use.

Four shRNA constructs were made for knocking down CD44 variant 9 (longest exon of CD44v7-10). Three sequences were generated by Extractor 4 computer program, and the fourth was from our prior publication[3]. Sequences were cloned into pSuper-RETRO (OligoEngine, Seattle) derived from pSuper[10].

2 × 10^6 ^293T cells were plated per 10 cm dish, and grown to 80% confluence in complete growth medium the next day. For luciferase-only and CD44s, 293T cells were transfected with Lentivector, packaging plasmid (psPAX2), and envelope plasmid (pMD2.G) using 3:1 PEI:DNA. For RNAi against CD44v7-10, the pSuper-RETRO with the construct was transfected similarly, but with 3 packaging vectors pJK3, pTAT2, and pVSVG, to produce retrovirus. After 48 h, supernatant was harvested and filtered with a 0.45 μm syringe filter. Incubation with viral supernatant for 2 days was used to infect the PC-3M cells. Cells were passaged into a 75 cm^2 ^flask and supplied with puromycin to final concentration of 1 μg/ml. A stable cell line was maintained by changing this selection medium every 3-7 days.

Plasmid pUHD10-3 that contained *Eco*R1 fragments for wild-type merlin cDNA was a gift of Dr. D. H. Gutmann of Washington University in St. Louis[11]. PCa cells were transfected using PEI in the same manner as the 293T cells. To alter p21-activated kinase-2 (PAK-2), on which merlin phosphorylation depends in DU145 cells[12], sense and antisense nucleotides were transfected as described[13] with PEI.

### Real Time Quantitative RT-PCR

To confirm altered CD44 expression, we used a primer + probe set that detects CD44 total, or a set that detects CD44v7-10 (Applied Biosystems, Foster City, CA) as described[14]. Detection of 18S ribosomal RNA was done simultaneously as a normalizer. TaqMan data were analyzed by the  method[14] to determine fold change in gene expression (untreated cells = 1.00). The ΔC_T _was taken as the difference between the CD44v or CD44 total C_T _and the 18S ribosomal RNA C_T_. The ΔΔC_T _was obtained using the mean ΔC_T _of untreated cells as calibrator. Each TaqMan result was compared to 1.00 using 2-tailed paired t-test.

### Migration and invasion assays

We used a population of PC-3M cells stably infected with lentivirus (for CD44s) or pSUPER-derived retrovirus (for CD44v RNAi). Invasion was assessed with triplicate 24-well Matrigel two-tier invasion chambers, and migration was assessed with control inserts, both with 8.0 μm pore diameter (Collaborative Biomedical Products, Bedford, MA)[4]. Untreated cells or those expressing a construct were seeded at 30,000 per well. Cells in the upper insert were in serum-free basal medium (RPMI 1640 with 0.1% BSA, 4 mM L-glutamine, 100 μg/mL each of penicillin G and streptomycin). The lower chamber contained chemoattractant medium consisting of 10% fetal bovine serum, 20% conditioned medium from subconfluent culture, and 70% complete medium. The incubation was carried out 24 h in 5% CO_2 _incubator at 37°C. The medium from the upper inserts, together with non-invading cells were removed off the upper Matrigel surface. The lower surface was fixed in methanol and stained with May-Grunwald stain (Sigma) according to manufacturer's protocol.

### Soft agar colony formation and growth assays

A bottom layer was formed using 2 ml complete medium with 1% agarose that was poured into each well of 6-well plates and solidified at 4°C. PC-3M cells with or without viral infection, 5000/well, were mixed in complete medium with 0.5% agarose and seeded as a top layer. The agarose was solidified at 4°C and then incubated at 37°C. On day 14, the colonies were stained with 1 ml of PBS containing 0.5 mg/ml of p-iodonitrotetrazolium violet (Sigma, St. Louis, MO). Only live cells convert this into a colored product. The total number of colonies from 20, 100× microscopic fields was counted. For growth assays, we seeded 30,000 cells per 6-well plate well and harvested and counted cells 3 days later by grid method.

### Cellular adhesion assays

Assays were carried out as described[15] using trypsinized confluent untreated or virally treated cells. Each test condition was set in 8 wells and each experiment repeated twice. 96-well black-edged clear flat bottom Costar plates (Cole-Parmer, Vernon Hills, IL) were coated with optimal concentrations of ligands[15] using 8 wells to test each one, at 37°C overnight. As controls, 8 wells were coated with 1 mg/ml BSA to measure baseline nonspecific binding. 1 × 10^6 ^cells suspended in 1 ml PBS were incubated with the dye BCECF-AM (Dojindo, Tokyo) for 15 min at 37°C. After two washes of the cells with PBS, cells with serum-free basal medium were added to plates at a density of 3 × 10^4 ^per well and incubated at 37°C for 90 min. Fluorescence intensities at 530 nm were measured using a Bio Tek FL-600 plate reader. Non-adherent cells were removed with 2 PBS washes. Fluorescence intensities with PBS in the wells were measured. Adhesion was calculated[15] as % cells bound = (100) fluorescence intensity post-wash/fluorescence intensity of total cells plated.

### Western blot analysis

Cultured cells were directly lysed in dishes using RIPA buffer (Upstate Biologicals, Lake Placid, NY) plus the protease inhibitor mini tablets (Applied Science, Indianapolis, IN). Protein concentration of the cell lysate was estimated by Bradford method. SDS-PAGE was performed on 25 μg sample/lane according to Laemmli method[16] using the NuPAGE system (Invitrogen, Carlsbad, CA). 10 μl of Kaleidoscope protein marker (Bio-Rad, Hercules, CA) was run in at least one lane. After electrophoresis for 2 hr, the protein was transferred to PVDF. Mouse monoclonal antibodies all used at 1 μg/mL were: to assess overexpressed CD44v7-10, CD44v10 antibody (Bender MedSystems, Burlingame, CA); CD44 total (standard + variant 156-3C11), CD44s (LabVision, Fremont, CA), and rabbit merlin and phosopho-merlin (Cell Signaling) were used at 1:1000. Anti-β-actin antibody (Sigma, St. Louis) was used at a dilution of 1:5,000. Membranes were washed 3 × 10 min in TBS with 0.1% Tween-20 (TBST) and 1:1000 dilution of goat anti-mouse IgG antibody labeled with biotin (Bio-Rad) or 1:1,250 goat anti-rabbit (Santa Cruz Biotechnology, Santa Cruz, CA) was added in 5% skim milk for 1 h. After washing membrane with 1× TBST, reactivity was detected using a chemiluminescent system (SuperSignal West Pico Substrate, Pierce Biotechnology, Rockford, IL). Each experimental run was conducted at least twice.

### Docetaxel Sensitivity Assays

Cells were seeded on a 96-well plate containing 5000 cells/well for 6 h. Docetaxel (Taxotere, Sanofi Aventis, Bridgewater, NJ) was administered to wells at doses from 0-50 nM. After 72 hours, MTS assay (CellTiter 96AQ, Promega, Madison, WI) for proliferation was performed and read on the Bio Tek. The plate was read at hourly intervals and the representative interval was chosen for each cell type so as to normalize intensity at time zero. Even so, the OD values at time zero ranged from 1.4 to 1.7 and had to be re-normalized before analysis. The assay was repeated twice with similar results.

### Xenograft Growth

Experiments were performed with the approval of the University of Colorado Denver Animal Care and Use Committee, 2007, and in conformity with national guidelines. A total of 32 NIH-III nude mice were used, including at least 7 per treatment group. 1.5 × 10^6 ^PC-3M-luciferase cells, CD44s-Separate cells, CD44s-Fusion cells, or CD44v7-10 RNAi cells, mixed 1:1 with Matrigel, were injected subcutaneously into the flank. Presence or absence of tumor 'take' was recorded, and tumor length, width, and height were measured daily. When tumor reached 2.0 cm in greatest dimension, mice were imaged on the IVIS 200 (Xenogen) to confirm persistent signal in all tumors, prior to mouse sacrifice.

### Statistical Analyses

For in vitro assays and mice growth, data were expressed as mean ± SD. The significance of differences among group means was tested by two-tailed 2-sample Student *t*-test. Statistical significance was set at p < 0.05.

## Results

### Establishment of altered CD44 expression

**Figure 2:**
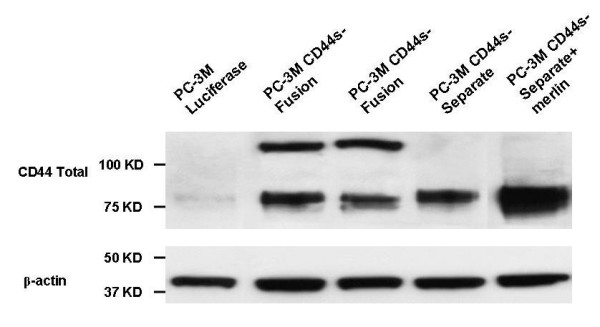
**Detection of total CD44, by monoclonal antibody in PC-3M cells. **The appearance of a predominant cleavage product at 75 kD (lane 1) rather than a higher molecular weight form, is expected based on our experience [1-4,14]. Re-expression of CD44s as a Fusion protein with luciferase produced a 137 kD form, with some residual 75-kD form, probably cleaved off after trypsinizing. The forced expression of CD44's intracellular binding partner merlin potentiated the increased CD44s (lane 5).

Western blot analyses for total human CD44 (Figure 2) in CD44s-Separate cells and CD44s-Fusion cells confirmed CD44s overexpression. Western blot analysis for CD44v7-10 was inadequate, since the antibodies we tested were not reliable. By Real Time RT-PCR, CD44 total was increased 1.80-fold in CD44-Fusion cells and 4.35-fold in CD44-Separate cells compared to untreated cells. CD44s re-expression as a fusion or separate protein did not affect CD44 variant. CD44v7-10 re-expression was reduced to 0.21 (79% silencing) in the 1522 viral RNAi construct. In this transfectant, knockdown was the best among 3 RNAi constructs, so we used it for all further work.

### Migration and invasion

**Figure 3:**
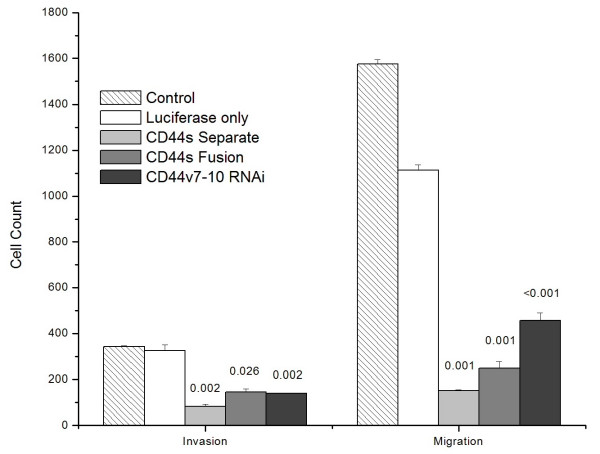
**The expression of CD44 as a Separate or Fusion protein, or enactment of CD44v7-10 RNAi, all significantly decreased invasion (Matrigel insert) and migration (control insert) of PC-3M cells. **p-values are indicated above bars.

PC-3M CD44s-Fusion cells, CD44s-Separate cells, and CD44v7-10 RNAi cells all displayed >50% decreases in both migration and invasion compared with the luciferase-only PC-3M control (Figure 3). Compared to the luciferase-only control cells, the CD44s-Separate cells had 86% less migration and 74% less invasion (p < 0.001). The CD44s-Fusion cells showed 78% decreased migration and 55% decreased invasion (p < 0.001).

### Attached cell growth and anchorage-independent growth

**Figure 4:**
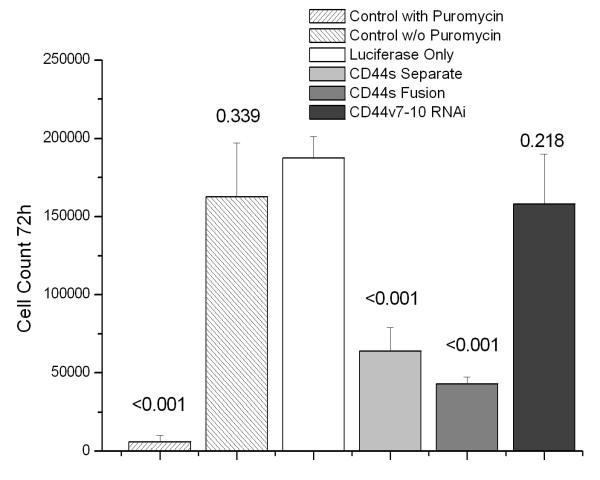
**The PC-3M-CD44s-Separate cells and CD44s-Fusion cells both showed decreased growth, assessed by growth assays of 30,000 cells after 3 days. **CD44v7-10 RNAi cells did not show decreased growth. Untreated cells were killed by puromycin (positive control). p-values are indicated above bars.

**Figure 5:**
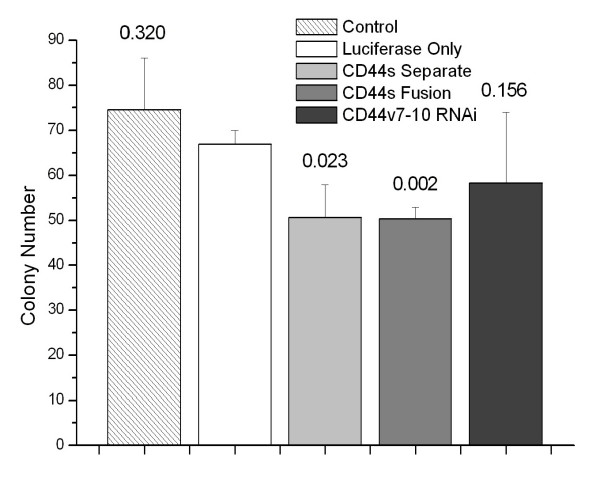
**Colony formation of PC-3M in soft agar after 14 days. **The CD44-Separate and CD44 Fusion cells grew significantly fewer colonies than controls, but CD44v7-10 RNAi cells did not. p-values are indicated above bars.

Compared to PC-3M-luciferase only cells, attached cell growth at 3 days was decreased in CD44s-Separate cells and CD44s-Fusion cells grown in puromycin-containing medium (both p < 0.001) but not in CD44v7-10 RNAi cells (p = 0.22, Figure 4). In soft agar assays, the same trends were evident, with 25% decreases in colonies formed from CD44s-Separate (p < 0.023) and CD44s-Fusion (p < 0.002) but not CD44v7-10 RNAi cells (p = 0.156) (Figure 5).

### Cell adhesion assay

**Figure 6:**
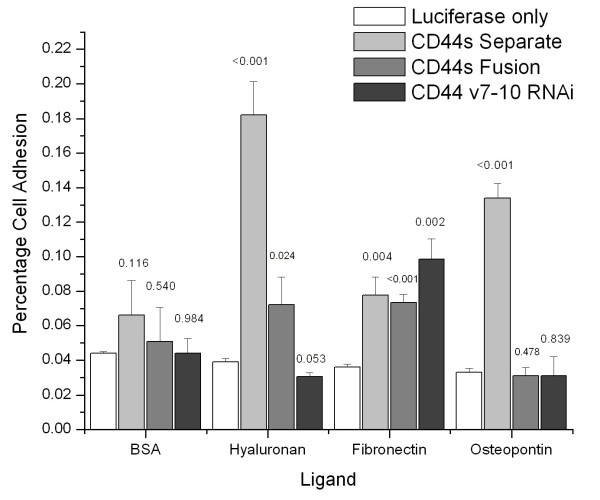
**Cell adhesion assay of PC-3M cells. **CD44s-Separate cells showed a 4-fold increase in hyaluronan binding and a 3-fold increase in osteopontin binding compared to luciferase-only cells. CD44s-Fusion cells showed a less marked but significant increase in hyaluronan binding, but not osteopontin binding. Fibronectin binding was slightly but significantly increased in all infected cells. p values are indicated above bars.

Increases of about 4-fold in percent cells binding to hyaluronan, and 3-fold in percent cells binding to osteopontin were noted with the CD44s-Separate cells (p < 0.001) compared to luciferase-only controls. The CD44s-Fusion cells showed a divergent response, with a minimal but significant increase in hyaluronan binding but no increase in osteopontin binding. Both CD44s infected cell lines and CD44v7-10 RNAi cells had slightly increased adhesion to fibronectin (Figure 6).

### Docetaxel sensitivity assay

**Figure 7:**
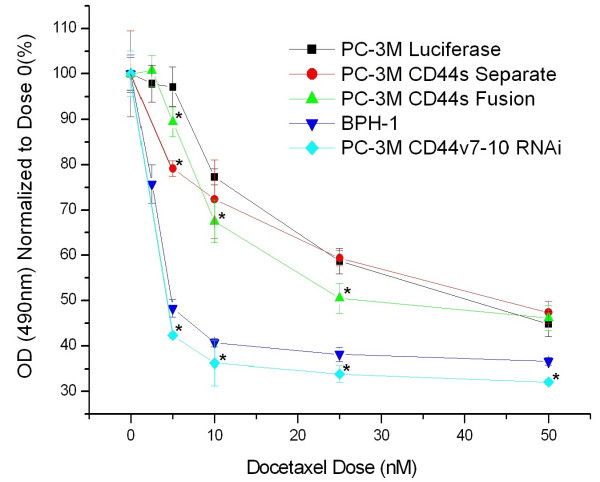
**Chemosensitivity by MTS assay. **PC-3M cells with CD44v7-10 RNAi were maximally sensitive to Docetaxel doses up to 50 nM. Moderate increases in sensitivity were noted after re-expression of CD44s as a Separate or Fusion protein. * indicates p < 0.05. As a control, benign BPH-1 prostate cells were also highly sensitive.

Increased sensitivity to Docetaxel doses of 5-50 nM was achieved in all treated cells, compared to PC-3M luciferase-only control cells. PC-3M CD44v7-10 RNAi cells were very sensitive (p < 0.05) across a 5-50 nM dose range. PC-3M CD44s-Fusion cells were significantly more sensitive at 5-25 nM, and PC-3M CD44s-Separate cells only at 5 nM. Benign BPH-1 cells were assessed as a very sensitive positive control (Figure 7).

### Xenograft growth

**Figure 8:**
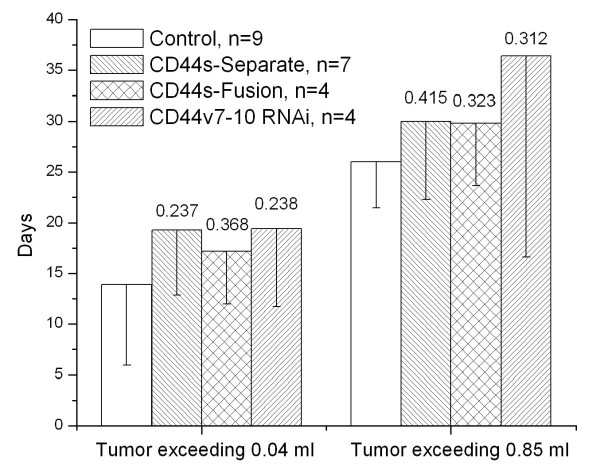
**Non-significant reductions in growth of subcutaneous xenografts in nude mice were noted in all treated cells, by Kaplan-Meier analysis. **Daily tumor volume was measured, and two cut points were chosen: 0.04 ml for formation of a palpable mass, and, based on the smallest tumor among mice with tumor take, 0.85 ml for a fully-formed tumor. n = number of mice with tumor take.

Tumor take for PC-3M control cells was 9/12 (75%), 7/8 (88%) for CD44s-Separate cells, but only 4/7 (57%) for CD44s-Fusion cells, and 4/7 (57%) for PC-3M CD44v7-10 RNAi cells. Tumor latency was defined as days until appearance of a 0.04 ml palpable mass. Appearance of a fully-formed mass was defined according to the smallest of the masses that developed among animals with tumor take, giving a set point of 0.85 ml. Mean number of days until these 2 points were reached was calculated for each cell type (Figure 8). There was no significant difference among the 4 groups by log-rank test (p = 0.89), but pairwise comparisons showed non-significant trends toward efficacy for all three therapies, particularly CD44v7-10 RNAi.

### Relationship of CD44 with Merlin

**Figure 9:**
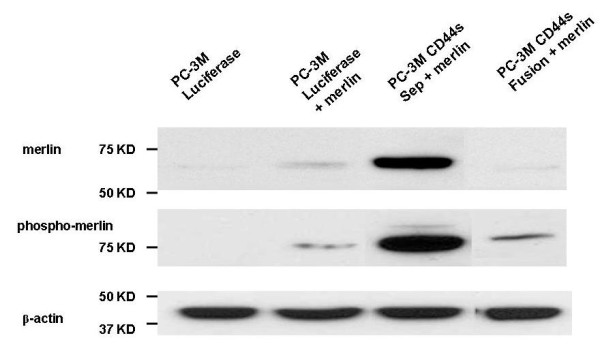
**Enforced expression of merlin increases total merlin and the predominant, lower molecular weight, hypophosphorylated form of p-merlin **(lane 2), and these effects are potentiated in virally-infected CD44s-Separate cells (lane 3). This effect is minimal in CD44s-Fusion cells (lane 4). Merlin overexpression also markedly increases CD44 total (Figure 2). Together, findings suggest that CD44s-merlin binding reduces degradation of both molecules.

Enforced expression of merlin increased CD44 total in CD44s-Separate cells (Figure 2). We also examined the degree of overexpression of merlin and phospho-merlin achievable in CD44s-Separate cells, compared to luciferase-only or CD44s-Fusion cells (Figure 9). Total merlin content was increased the most in CD44s-Separate cells. Immunoblotting for phospho-merlin, confirmed by the manufacturer to be phospho-specific to P-Ser 518, demonstrated a great increase in a band representing hypophosphorylated, 75 kD merlin in CD44s-Separate cells. The luciferase-only cells and CD44s-Fusion cells, in contrast, had less 75-kD, hypophosphorylated form as well as a band for the 85-kD form, reflecting phosphorylation at multiple sites.

### Dependence of Phospho-merlin on PAK2

**Figure 10:**
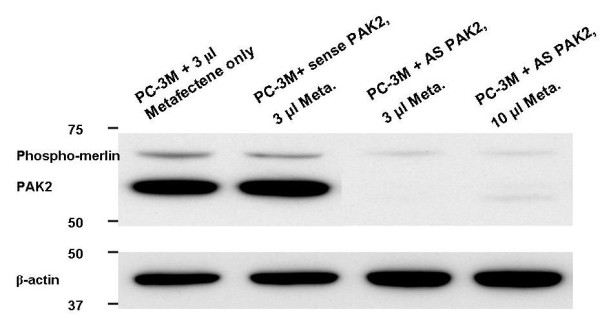
**Antisense inhibition of PAK2 causes a decrease in p-merlin in PC-3M cells. **PAK2 is an enzyme that phosphorylates (and activates) merlin; thus, the preferentially expressed hypophosphorylated form of merlin in PC-3M cells depends on PAK2, as it does in DU145 cells[12].

To determine whether expression of the phospho-merlin were dependent on PAK2, as is the case in DU145 cells[12], PC-3M cells were transfected with merlin and with PAK2 antisense oligonucleotide or a sense sequence. Antisense against PAK2 in luciferase-only PC-3M cells was effective, and profoundly decreased phospho-merlin (Figure 10).

## Discussion

We have shown that in aggressive PC-3M PCa cells, re-expression of CD44s greatly reduced invasion, migration, and tumor cell proliferation, suggesting a number of functional effects in PCa and possibly certain other cancers such as bladder, for which CD44s functions as a tumor suppressor.

In vitro, PCa growth and soft agar colony formation were inhibited more effectively by CD44s-Separate than any other treatment. This finding is consistent with Orian-Rousseau et al., who found, using rat pancreas cells, that overexpression of the CD44s cytoplasmic tail was able to inhibit pro-growth Met-Ras-ERK signaling[17]. It also explains why CD44s-Fusion was not associated with increased merlin, since the C-terminus luciferase would render inaccessible the amino acids 292-300 that bind merlin (and ERM proteins). Furthermore, this blockage of binding in CD44s-Fusion cells would explain the lack of increase in growth-inhibiting hypophosphorylated merlin after merlin transfection (Figure 8). With colon cancer cells, conversely, CD44s had an anti-apoptotic effect in vivo, yet CD44s knockdown did not affect proliferation in vitro[15]. Lastly, two recent papers concern neuroendocrine cells in prostate cancer. The studies used the IM7 rat monoclonal antibody against HCAM/PgP, and the DF1485 mouse monoclonal antibody against HCAM/PgP. Both of these antibodies are raised against lymphocyte membrane and product information shows they detect a 90-kD protein consistent with CD44s. When we used the DF1485, it was to detect CD44s [14]. These antibodies do not detect the epithelial CD44v8-10 or the v7-10 of non-neuroendocrine prostate cancer. In neuroendocrine carcinoma, a rare prostate tumor composed entirely of neuroendocrine cells, CD44s immunostaining was very strong[18], whereas in non-neuroendocrine carcinoma only scattered cells were CD44s-positive as shown previously[5,6] and those same cells marked as neuroendocrine cells[18,19]. Because CD44s marks prostatic stem cells (basal cells)[1], neuroendocrine carcinoma may be derived from them, and CD44s may exert an influence different from its role in non-neuroendocrine cancer cells, detailed here.

Invasion and migration were strongly inhibited through CD44s overexpression, and more weakly but significantly through knockdown of CD44v7-10, as we showed previously[3]. Interpretation of the latter finding could be limited, because although the luciferase-only control cells controlled for a nonspecific effect of viral infection, PCa infection to express CD44v7-10 sense strand as a control for off-target effect was not performed. Invasion was assessed through Matrigel membranes, which have no hyaluronan but do have fibronectin, consistent with Gao's observation that CD44s suppression of invasion and metastasis does not rely on hyaluronan binding[20].

Altered CD44 expression also affected ligand binding, with increased hyaluronan binding in the CD44s-Separate cells compared to CD44s-Fusion. As an explanation, the mature form of human CD44s lacks the protein product of exon 19 but contains most of exon 20 at its cytoplasmic, C-terminus. A functional C-terminus is required not only for CD44 binding to ankyrin[21] but also for hyaluronan binding by the "link" domain at the N-terminus[22] which involves oligomerization[23], and for formation of the pericellular matrix[22].

We previously determined that CD44v7-10 was functionally significant for PCa invasion[3] and is increased by calcitonin, a pro-growth paracrine hormone[14]. CD44 variants are known to oligomerize, causing pro-growth signaling[23], but in the current studies, the CD44v7-10 did not appear significant for in vitro, anchorage-dependent or independent growth or xenograft growth. CD44v7-10 RNAi did not affect merlin or phospho-merlin, consistent with this lack of an observed effect on growth. However, RNAi enforced against CD44v7-10 was most effective for chemosensitization of PC-3M cells. Docetaxel sensitivity by MTS assay was maximal in these cells. This result affirms an anti-apoptotic effect of CD44 variant forms in cancer. Jurkat T-cell leukemia cells, for instance, are made refractory to Fas-mediated apoptosis by their expression of CD44v6 and v9 isoforms [24], and CD44v7 in rat adenocarcinoma cells confers apoptosis resistance by tumor cell-matrix cross-talk [25]. Growth is also stimulated by CD44 variants since they uniquely multimerize with and facilitate the hepatocyte growth factor-Met interaction, resulting in ERK activation[17].

CD44s-Separate cells and CD44v7-10 RNAi cells, despite having dramatic anti-growth or pro-chemosensitivity properties in vitro, showed only non-significant trends toward slower mouse subcutaneous xenograft growth. Altered expression of other cell adhesion molecules has been described to cause divergent in vitro and in vivo effects. For example, in thyroid cancer, expression of CEACAM1, a molecule also involved in both adhesion and cell signaling for growth pathways, facilitated invasion but slowed growth of tumor xenografts[26]. However, two limitations of our study are sample size (n = 7 or 8 per group with altered CD44), and not having tested orthotopic xenografts. Orthotopic grafts, in a different stromal and matrix metalloprotease microenvironment, are often slower-growing, non-metastatic, and respond to certain therapies that subcutaneous ones do not[27]. We tried to overcome this limitation by use of Dunning rat PCa cells, in which subcutaneous grafts can metastasize to lungs[18] but re-expression of human CD44s as a fusion or separate protein could not be documented on western blot analysis, and human CD44v was absent (data not shown), possibly because a few CD44 bases differ between human and rat. Thus, our use of the subcutaneous approach may have precluded assessment of relevant therapeutic effects.

Merlin, a downstream signaling effector of CD44, is phosphorylated under growth-promoting conditions and dephosphorylated under growth-inhibiting conditions including CD44-hyaluronan binding[11,28]. Dephosphorylation inactivates it by a conformational change to a circularized form. In PC-3M cells, using a phospho-specific antibody, we detected a band representing hypophosphorylated merlin. The band was at 70 kD, as opposed to the expected 75 kD, and was dependent on PAK2, as shown by knockdown of PAK2 (Figure 10). This band was greatly strengthened in CD44s-Separate cells, suggesting it might facilitate the increased hyaluronan binding of CD44s-Separate cells. However, fully phosphorylated, active merlin inhibits CD44-hyaluronan binding[28] so our detection of this form suggests that in PC-3M cells, CD44s stabilizes merlin in a hypophosphorylated state that is growth-inhibitory yet permits CD44s-hyaluronan binding. The CD44s-Fusion cells have their cytoplasmic C-terminus blocked by the attached luciferase, correlating with less phospho- and total merlin, and less hyaluronan binding. The increased binding to osteopontin by CD44s-Separate, but not CD44s-Fusion, suggests that a functional C-terminus may also faciliate osteopontin binding. Interestingly, the increased osteopontin adhesion in CD44s-Separate cells disagrees with a previous conclusion that CD44 variants, but not CD44s, facilitated osteopontin binding to allow migration[29]; however, these data were based on rat pancreatic carcinoma, rat fibrosarcoma, and mouse melanoma cells, and pertained to CD44v4-7, not CD44v7-10.

Horiguchi et al. [12] found that silencing of CD44 dephosphorylates merlin in benign cells, and that merlin was strongly expressed and constitutively phosphorylated in DU145 PCa cells but weak to absent in LNCaP, PC3, 22RV1, and LAPC-4. Silencing total CD44 in DU145 cells did not affect merlin and phospho-merlin. However, our enforced merlin expression in PC-3M-luciferase cells resulted in increased CD44s. Enforced CD44s-Separate, in turn, increased the hypophosphorylated merlin compared to the minimal, mostly hyperphosphorylated amount in PC-3M-luciferase cells (Figure 2). This may reflect mutual stabilization of CD44s bound to merlin.

Finally, we considered the attractive prospect of altering both CD44s and CD44v7-10. It was not possible, under our system, to establish doubly-infected cells for CD44s and CD44v7-10-RNAi, because both rely on puromycin for selection, and flow cytometry for luciferase is not feasible.

## Conclusions

The stable re-expression of CD44s reduces PCa growth and invasion in vitro, and causes a non-significant growth reduction in the subcutaneous xenograft model. When the C-terminus of CD44s is fused to another protein, some phenotypic changes are lessened. Finally, CD44v7-10, although it was not functionally significant for growth, may be a target for chemosensitization. These findings suggest the potential for CD44 alterations in gene therapy, perhaps in conjunction with enforced alterations of the expression of other molecules.

## Abbreviations

CD44: Cell determinant 44; CD44s: CD44 standard; CD44v: CD44 variant; PEI: polyethyleneimine; RNAi: RNA interference.

## Competing interests

The authors declare that they have no competing interests.

## Authors' contributions

All authors performed experiments; KY performed statistics; KAI and KY wrote the paper. All authors read and approved the final manuscript.

## Pre-publication history

The pre-publication history for this paper can be accessed here:

http://www.biomedcentral.com/1471-2407/10/16/prepub
